# Early posttransplant rituximab use in kidney transplant recipients with preexisting donor-specific antibodies

**DOI:** 10.1080/0886022X.2026.2620179

**Published:** 2026-01-25

**Authors:** Junji Yamauchi, Katalin Fornadi, Divya Raghavan, Duha Jweehan, Suayp Oygen, Silviana Marineci, Ann Pole, Dharmendra Jain, Eszter Lazar-Molnar, Miklos Z. Molnar

**Affiliations:** ^a^ Department of Internal Medicine, Division of Nephrology & Hypertension, Spencer Fox Eccles School of Medicine at the University of Utah, Salt Lake City, UT, USA; ^b^ Department of Surgery, Division of Transplantation and Advanced Hepatobiliary Surgery, Spencer Fox Eccles School of Medicine at the University of Utah, Salt Lake City, UT, USA; ^c^ Histocompatibility and Immunogenetics Laboratory, University of Utah Health, Salt Lake City, UT, USA; ^d^ Department of Pathology, Spencer Fox Eccles School of Medicine at the University of Utah, Salt Lake City, UT, USA

**Keywords:** Kidney transplantation, donor specific antibodies, rituximab, rejection, infection

## Abstract

The presence of pretransplant anti-human leukocyte antigen (HLA) donor-specific antibodies (DSAs) is still a significant barrier to successful kidney transplantation, as it increases the risk of rejection and graft failure. Rituximab (anti-CD20 antibody) has been administered in hopes of suppressing DSA production and rejection in those with preformed DSAs. However, existing studies report conflicting outcomes, underscoring the need for more data to guide clinical practice. Thus, we evaluated the efficacy of early posttransplant rituximab administration in a cohort of kidney transplant recipients with pretransplant anti-HLA DSAs. In this retrospective study of 77 patients, we compared 1-year transplant outcomes between patients treated with and without rituximab for pretransplant anti-HLA DSAs. Infectious complications tended to occur more often in the rituximab group (BK polyomavirus DNAemia >10,000 copies/mL, 3 [19%] vs. 8 [13%]; quantifiable cytomegalovirus DNAemia, 8 [50%] vs. 19 [31%]; infection requiring hospitalization, 5 [31%] vs. 11 [18%]), but none of these differences reached statistical significance. The incidence of biopsy-proven rejection (2 [13%] vs. 12 [20%]) and high plasma donor-derived cell-free DNA (2 [18%] vs. 12 [27%]) tended to be more frequent in the no-rituximab group, but none of these reached statistical significance. Preexisting DSA persisted or recurred in 44% of the patients that received rituximab, and in 46% of patients who did not receive rituximab. Similarly, *de novo* DSA occurred in 31% of those who received rituximab versus in 25% of those who did not. Rituximab administration did not result difference in graft and patient survival or rejection rates or recurrence of preexisting DSA.

## LAY SUMMARY

Our single center study examined whether giving rituximab shortly after kidney transplantation helps reduce rejection in patients who already had donor-specific antibodies before transplant. We compared outcomes over one year between patients who received rituximab and those who did not, focusing on antibody recurrence, rejection episodes, kidney function, and infections. Rituximab-treated patients had similar kidney function and rates of donor-specific antibody recurrence compared with untreated patients, with a trend toward fewer rejection events occurring early after transplant. However, patients who received rituximab showed a tendency toward higher rates of viral infections, although these differences were not statistically significant. Overall, early post-transplant rituximab did not clearly reduce antibody recurrence or rejection in this setting and should be weighed carefully against its potential infectious risks.

## Introduction

The presence of preexisting donor-specific antibodies (DSA) represents a significant barrier to successful kidney transplantation, as it increases the risk of rejection and graft failure [1–3]. Rituximab, an anti-CD20 monoclonal antibody, has been administered to kidney recipients with preexisting DSA, either before or early after transplant, aiming to suppress DSA production and rejection. Rituximab is also used in kidney transplant desensitization as an anti‑CD20 B‑cell–depleting agent, aimed not at acutely removing DSA but at blunting memory B‑cell and germinal center responses to reduce DSA rebound and new alloantibody formation after antibody removal [4–8]. In multimodal protocols (typically with intravenous immunoglobulin ± plasmapheresis/immunoadsorption), rituximab helps lower overall human leucocyte antigen (HLA) antibody levels, stabilizes DSA kinetics, and facilitates conversion of positive crossmatches to negative, thereby increasing access to transplantation for highly sensitized candidates [4–6]. Controlled data suggest rituximab clearly improves DSA kinetics (less rebound, greater mean fluorescence intensity (MFI) reduction) but its incremental effect on ‘hard’ outcomes like antibody-mediated rejection incidence and long‑term graft survival beyond similar regimens without rituximab is only partially defined [8]. Use of rituximab in desensitization is also consistently associated with higher infectious risk—particularly serious bacterial and viral infections—so its benefits must be balanced against added immunosuppressive toxicity and managed with careful prophylaxis and immunosuppression tailoring [9].

However, published data on its efficacy remain limited for transplantation with negative crossmatch and preexisting DSA, and report conflicting outcomes [6,8,10–14]. We therefore investigated the efficacy and safety of early post-transplant rituximab administration in these patients. We hypothesized that rituximab administration reduces the probability of antibody-mediated rejection by decreasing the risk of preexisting DSA recurrence after transplantation while not increasing the risk of infectious complications.

## Methods

We retrospectively reviewed our adult kidney-alone transplant recipients with pre-transplant anti-HLA DSA between 01/01/2019 and 05/31/2024 (Institutional Review Board approval number: IRB_00162331). We included recipients with detectable DSA at the time of transplant with normalized MFI >2000 set by the laboratory, and those with a history of DSA that were below detection limit at the time of transplant (historic DSA). Recurrence of preexisting DSA was defined as positive if DSA present at the time of transplant (>2000 MFI), or present historically but below cutoff at the time of transplant was detected within one year after transplantation (MFI > 2000). *De novo* DSA was defined positive if detected within one year after transplantation with MFI > 2000 but not detected prior to transplant historically, or at the time of transplantation.

HLA typing was conducted using molecular methods, involving genomic DNA amplification and sequence-based typing and/or the sequence-specific oligonucleotide probing method. T-cell and B-cell crossmatches were performed *via* flow cytometry, with results classified as negative, weakly positive, or positive based on MFI thresholds of <3, 3–4, or >4 standard deviations above the negative control, respectively. HLA antibody testing against HLA-A, -B, -C, -DRB1, -DRB3, -DRB4, -DRB5, -DQB1, -DQA1, -DPB1, and -DPA1 was performed using a Luminex-based single antigen bead array (LabScreen, One Lambda/ThermoFisher Scientific) following serum heat-treatment (56 °C/30 min) to eliminate any possible complement interference in the assay.

Prior to transplantation, patients were tested for HLA antibodies quarterly, and at the time of transplant to define baseline DSA. Post-transplant DSA monitoring was performed at 1, 2, 3, 6, and 12 months and when clinically indicated at the discretion of the transplant nephrologist. Donor-derived cell-free DNA (Allosure^™^, Prospera^™^, or Viracor TRAC^™^) in plasma was checked at 1, 2, 3, 4, 5, 6, 9 and 12 months. Donor-derived cell-free DNA >1.0% was considered positive for Allosure and Prospera, and >0.7% for Viracor TRAC. Kidney biopsies were conducted only when clinically indicated and diagnosed according to the Banff 2019 classification [7].

One-year transplant outcomes were compared between recipients treated with and without rituximab. Rituximab was administered as a single dose of 375 mg/m^2^ of body surface area within 14 days posttransplant. Kidney biopsies were performed only when clinically indicated (based on elevated donor derived cell-free DNA, elevated serum creatinine, new onset or worsening proteinuria, hematuria, new onset or worsening proteinuria, clinical concern of recurrent glomerulonephritis) and were diagnosed according to the Banff 2019 classification. Given the small sample size, we did not evaluate statistical significance, focusing instead on descriptive analysis. This study was approved by the University of Utah Institutional Review Board (IRB_00162331), which granted an exemption from informed consent.

## Results

**Figure 1.**
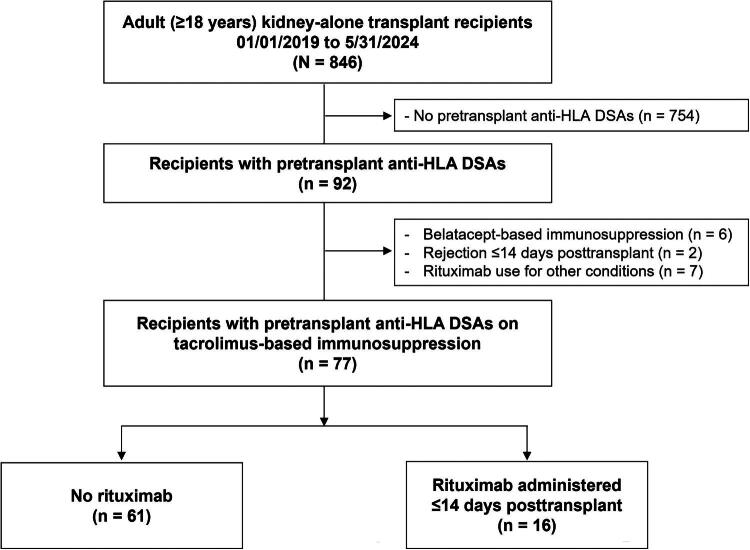
Study flowchart. anti-HLA DSAs: anti-human leukocyte antigen donor-specific antibodies.

A total of 77 recipients (16 with and 61 without rituximab) were included in the analysis (Figure 1). Patient characteristics are summarized in Table 1. The median number of HLA-A/B/DR mismatches was 4 (interquartile range, 3–5) in both groups. Calculated panel reactive antibody of >80% occurred more often in the rituximab group (8 [50%] vs. 15 [25%]). Flow cytometric T crossmatches were more commonly positive in the group that received rituximab. All recipients received lymphocyte-depleting antibody induction (anti-thymocyte globulin or alemtuzumab) followed by tacrolimus/mycophenolate/prednisone maintenance immunosuppression. In the rituximab group, 4 (25%) patients underwent a session of plasma exchange and administration of intravenous immune globulin immediately prior to transplantation. Rituximab was given at a median of 8 (7–9) days posttransplant. Other baseline characteristics were comparable, except for higher donor terminal serum creatinine (mean ± standard deviation, 1.1 ± 0.5 vs. 0.9 ± 0.4 mg/dL) and longer cold ischemia time (20.3 ± 10.1 vs. 11.9 ± 7.7 h) in the rituximab group.

**Table 1.** Patient characteristics.

	Total	Rituximab (–)	Rituximab (+)	
Characteristic	*N* = 77	*N* = 61	*N* = 16	*p*-value
Recipients				
Recipient age (years), mean (SD)	47 (15)	47 (15)	49 (15)	0.70
Recipient sex				0.41
Female	40 (52%)	30 (49%)	10 (63%)	
Male	37 (48%)	31 (51%)	6 (38%)	
Recipient race				0.62
White	48 (62%)	38 (62%)	10 (63%)	
Black	1 (1%)	1 (2%)	0 (0%)	
Hispanic	21 (27%)	15 (25%)	6 (38%)	
Asian	1 (1%)	1 (2%)	0 (0%)	
Native Hawaiian/other Pacific Islander	6 (8%)	6 (10%)	0 (0%)	
Body mass index, mean (SD)	27.5 (5.0)	27.7 (5.2)	26.5 (4.3)	0.41
History of diabetes	22 (29%)	18 (30%)	4 (25%)	1.00
Dialysis duration				0.37
Preemptive	12 (16%)	11 (18%)	1 (6%)	
< =1 year	11 (14%)	7 (11%)	4 (25%)	
1–3 years	21 (27%)	17 (28%)	4 (25%)	
3–5 years	21 (27%)	18 (30%)	3 (19%)	
>5 years	12 (16%)	8 (13%)	4 (25%)	
Cause of kidney failure				0.98
Diabetes	16 (21%)	13 (21%)	3 (19%)	
Hypertension	9 (12%)	7 (11%)	2 (13%)	
Glomerulonephritis	17 (22%)	14 (23%)	3 (19%)	
Cystic disease	11 (14%)	9 (15%)	2 (13%)	
Others	24 (31%)	18 (30%)	6 (38%)	
Prior kidney transplant	16 (21%)	11 (18%)	5 (31%)	0.30
Cytomegalovirus antibody				0.79
D negative/R negative	14 (18%)	12 (20%)	2 (13%)	
D any/R positive	47 (61%)	37 (61%)	10 (63%)	
D positive/R negative	16 (21%)	12 (20%)	4 (25%)	
HLA mismatch (A, B, DR), median (IQR)	4 (3–5)	4 (3–5)	4 (3–5)	0.14
HLA mismatch (A, B, C, DRB1, DQB1, and DPB1), median (IQR)	8 (6–9)	8 (6–9)	7 (5–9)	0.07
A mismatch				0.58
0	9 (12%)	8 (13%)	1 (6%)	
1	40 (52%)	30 (49%)	10 (63%)	
2	28 (36%)	23 (38%)	5 (31%)	
B mismatch				0.20
0	7 (9%)	4 (7%)	3 (19%)	
1	29 (38%)	22 (36%)	7 (44%)	
2	41 (53%)	35 (57%)	6 (38%)	
C mismatch				0.02
0	9 (12%)	4 (7%)	5 (31%)	
1	36 (47%)	30 (49%)	6 (38%)	
2	32 (41%)	27 (44%)	5 (31%)	
DRB1 mismatch				0.89
0	13 (17%)	10 (16%)	3 (19%)	
1	36 (47%)	28 (46%)	8 (50%)	
2	28 (36%)	23 (38%)	5 (31%)	
DQB1 mismatch				0.69
0	15 (19%)	13 (21%)	2 (13%)	
1	42 (55%)	32 (53%)	10 (63%)	
2	20 (26%)	16 (26%)	4 (25%)	
DPB1 mismatch				0.44
0	11 (14%)	8 (13%)	3 (19%)	
1	31 (40%)	23 (38%)	8 (50%)	
2	35 (45%)	30 (49%)	5 (31%)	
Calculated PRA at transplant (%), median (IQR)	8 (0–91)	0 (0–71)	54 (0–99)	0.12
Calculated PRA at transplant				0.059
< =20%	43 (56%)	35 (57%)	8 (50%)	
>20–80%	11 (14%)	11 (18%)	0 (0%)	
>80%	23 (30%)	15 (25%)	8 (50%)	
Flow cytometric T-cell crossmatch				0.006
Negative	73 (95%)	60 (98%)	13 (81%)	
Positive	4 (5%)	1 (2%)	3 (19%)	
Flow cytometric B-cell crossmatch*				0.05
Negative	75 (99%)	60 (100%)	15 (94%)	
Positive	1 (1%)	0 (0%)	1 (6%)	
Pretransplant plasma exchange	4 (5%)	0 (0%)	4 (25%)	0.001
Pretransplant intravenous immunoglobulin	4 (5%)	0 (0%)	4 (25%)	0.001
Induction immunosuppression				0.44
Anti-thymocyte globulin	66 (86%)	51 (84%)	15 (94%)	
Alemtuzumab	11 (14%)	10 (16%)	1 (6%)	
Days from transplant to rituximab administration	8 (7–9)	–	8 (7–9)	–
Donors				
Donor type				0.72
Living donor	15 (19%)	13 (21%)	2 (13%)	
Deceased donor	62 (81%)	48 (79%)	14 (88%)	
Donor age (years)	36 (15)	37 (15)	32 (10)	0.20
Donor sex				1.00
Female	36 (47%)	29 (48%)	7 (44%)	
Male	41 (53%)	32 (52%)	9 (56%)	
Terminal serum creatinine (mg/dL), mean (SD)	1.0 (0.4)	0.9 (0.4)	1.1 (0.5)	0.049
Kidney Donor Profile Index, mean (SD)	35 (24)	37 (26)	28 (18)	0.21
Donor kidney on-pump	58 (75%)	47 (77%)	11 (69%)	0.52
Cold ischemia time (hours), mean (SD)	13.6 (8.9)	11.9 (7.7)	20.3 (10.1)	<0.001

Values are expressed as mean (standard deviation, SD), median (interquartile range, IQR), or number (%). *P*-values were calculated using t-tests or Wilcoxon rank-sum tests for continuous variables, and Fisher’s exact tests for categorical variables.

PRA: panel reactive antibody.

* One patient’s B cell crossmatch result was indeterminate secondary to lack of B cells.

Table 2 summarizes 1-year outcomes. There were no deaths or graft failures in the rituximab group, while the no-rituximab group reported 1 (2%) death and 1 (2%) graft failure. The mean estimated glomerular filtration rate at 1 year was similar (67 ± 19 vs. 64 ± 22 mL/min/1.73m^2^) between the two groups. Infectious complications tended to occur more often in the rituximab group (BK polyomavirus DNAemia >10,000 copies/mL, 3 [19%] vs. 8 [13%]; quantifiable cytomegalovirus DNAemia, 8 [50%] vs. 19 [31%]; infection requiring hospitalization, 5 [31%] vs. 11 [18%]), but none of these differences reached statistical significance. The incidence and severity of neutropenia was also similar between two groups. The incidence of biopsy-proven rejection (2 [13%] vs. 12 [20%]) and high plasma donor-derived cell-free DNA (2 [18%] vs. 12 [27%]) tended to be more frequent in the no-rituximab group, but none of these reached statistical significance. In the rituximab group, there was acute T-cell mediated rejection in a patient with current DSA (6%) and mixed rejection in a patient with historic DSA (6%); both rejections occurred within 3-month post-transplant. The no-rituximab group exhibited 4 (7%) borderline, 1 (2%) acute T-cell mediated, 5 (8%) active antibody-mediated, and 4 (7%) mixed rejections; most rejections (83%) occurred after 3-month post-transplant.

**Table 2.** One-year kidney transplant outcomes.

	Total	Rituximab (–)	Rituximab (+)	
Outcome	*N* = 77	*N* = 61	*N* = 16	*p*-value
Survival and Graft Function:				
Death within 12 months	1 (1%)	1 (2%)	0 (0%)	1.00
Cause of death within 12 months	Disseminated CMV and pneumonia	Disseminated CMV and pneumonia	N/A	1.00
Graft failure within 12 months	1 (1%)	1 (2%)	0 (0%)	1.00
Cause of graft loss within 12 months: Non resolving Acute tubular necrosis	1 (1%)	1 (2%)	0 (0%)	1.00
Serum creatinine at 12 months (mg/dL), mean (SD)	1.4 (0.6)	1.4 (0.5)	1.3 (0.9)	0.88
12-month eGFR (mL/min/1.73m2), mean (SD)	65 (21)	64 (22)	67 (19)	0.62
Infectious complication:				
**Plasma BK polyomavirus DNA >10,000 copies/mL**	**11 (14%)**	**8 (13%)**	**3 (19%)**	**0.69**
BK polyomavirus nephropathy on biopsy	5 (6%)	3 (5%)	2 (13%)	0.28
**Cytomegalovirus viremia (quantifiable plasma DNA)**	**27 (35%)**	**19 (31%)**	**8 (50%)**	**0.24**
**Infection requiring hospitalization**	**16 (21%)**	**11 (18%)**	**5 (31%)**	**0.30**
**Neutropenia (WBC < 4,000/µl within 12 months)**	**64 (83%)**	**49 (80%)**	**15 (94%)**	**0.20**
Absolute neutrophil count grade I (1,000–1,499/µl within 12 months)	13 (17%)	11 (18%)	2 (13%)	0.60
Absolute neutrophil count grade II (500–999/µl within 12 months)	17 (22%)	12 (20%)	5 (31%)	0.32
Absolute neutrophil count grade III (100–499/µl within 12 months)	14 (18%)	11 (18%)	3 (19%)	0.95
Absolute neutrophil count grade IV (<100/µl within 12 months)	2 (3%)	2 (3%)	0 (0%)	0.46
Rejection^a^				
Kidney biopsy within 12 months	32 (42%)	24 (39%)	8 (50%)	0.57
**Any rejection (including borderline)**	**14 (18%)**	**12 (20%)**	**2 (13%)**	**0.72**
Time to any 1st rejection (including borderline)				0.066
< =3 months	4 (29%)	2 (17%)	2 (100%)	
>3 months	10 (71%)	10 (83%)	0 (0%)	
Any rejection (excluding borderline)	12 (16%)	10 (16%)	2 (13%)	1.00
Time to any 1st rejection (excluding borderline)				0.045
< =3 months	3 (25%)	1 (10%)	2 (100%)	
>3 months	9 (75%)	9 (90%)	0 (0%)	
T-cell mediated rejection				0.36
No	71 (92%)	56 (92%)	15 (94%)	
Yes	2 (3%)	1 (2%)	1 (6%)	
Borderline	4 (5%)	4 (7%)	0 (0%)	
T-cell mediated rejection grade				0.22
Borderline	4 (36%)	4 (44%)	0 (0%)	
Grade IA	3 (27%)	3 (33%)	0 (0%)	
Grade IIA	3 (27%)	1 (11%)	2 (100%)	
Grade III	1 (9%)	1 (11%)	0 (0%)	
Antibody mediated rejection	5 (6%)	5 (8%)	0 (0%)	0.58
Mixed rejection	5 (6%)	4 (7%)	1 (6%)	1.00
Microvascular inflammation (Banff g + ptc scores), median (IQR)	0 (0–2)	0 (0–2)	0 (0–2)	0.52
Microvascular inflammation (Banff g + ptc scores)				0.86
0	18 (56%)	13 (54%)	5 (63%)	
1	3 (9%)	2 (8%)	1 (13%)	
> =2	11 (34%)	9 (38%)	2 (25%)	
Plasma dd-cfDNA				
Name of dd-cfDNA test performed				0.10
Allosure	37 (66%)	31 (69%)	6 (55%)	
Prospera	14 (25%)	12 (27%)	2 (18%)	
Viracor TRAC	5 (9%)	2 (4%)	3 (27%)	
Number of dd-cfDNA measurement, median (IQR)	6 (4–7)	6 (4–7)	5 (3–7)	0.16
Positive dd-cfDNA (>1% for Allosure/Prospera, >0.7% for Viracor TRAC)	14 (25%)	12 (27%)	2 (18%)	0.71
Averaged dd-cfDNA (%), median (IQR)^b^	0.26 (0.13–0.50)	0.33 (0.15–0.52)	0.15 (0.04–0.37)	0.17
**Preexisting DSA**				
**Recurring any preexisting DSA positive within 12 months**				**0.88**
** No**	**42 (55%)**	**33 (54%)**	**9 (56%)**	
** Yes**	**35 (45%)**	**28 (46%)**	**7 (44%)**	
Recurring any preexisting class I DSA positive within 12 months				0.28
No	54 (70%)	41 (67%)	13 (81%)	
Yes	23 (30%)	20 (33%)	3 (19%)	
Recurring any preexisting class II DSA positive within 12 months				0.33
No	64 (83%)	52 (85%)	12 (75%)	
Yes	13 (17%)	9 (15%)	4 (25%)	
***De novo* DSA**				
***De novo* DSA class I and/or II development within 12 months**				**0.59**
** No**	**57 (74%)**	**46 (75%)**	**11 (69%)**	
** Yes**	**20 (26%)**	**15 (25%)**	**5 (31%)**	
*De novo* DSA class I development within 12 months				0.24
No	65 (84%)	53 (87%)	12 (75%)	
Yes	12 (16%)	8 (13%)	4 (25%)	
*De novo* DSA class II development within 12 months				0.95
No	63 (82%)	50 (82%)	13 (81%)	
Yes	14 (18%)	11 (18%)	3 (19%)	

Values are expressed as mean (standard deviation, SD), median (interquartile range, IQR), or number (%). *P*-values were calculated using t-tests or Wilcoxon rank-sum tests for continuous variables, and Fisher’s exact tests for categorical variables.

^a^ Rejection was diagnosed according to the Banff 2019 classification.

^b^ The average of dd-cfDNA values measured within 12 months posttransplant.

dd-cfDNA, donor-derived cell-free DNA; DSA, donor-specific antibody.

The post-transplant preexisting and *de novo* DSA incidence was similar between the two groups in the first year after transplantation (Table 2). Preexisting DSA persisted or recurred in 44% of the patients that received rituximab, and in 46% of patients who did not receive rituximab. Similarly, *de novo* DSA occurred in 31% of those who received rituximab versus in 25% of those who did not.

## Discussion

In this retrospective single-center study, we evaluated the efficacy and safety of early post-transplant rituximab administration in kidney transplant recipients with preexisting donor-specific antibodies and mostly negative crossmatch at the time of transplantation. Our findings suggest that a single dose of rituximab administered early after transplant did not result in a clear reduction in the incidence of preexisting DSA recurrence, *de novo* DSA development, or biopsy-proven rejection within the first year after transplantation when compared with a similar cohort managed without rituximab. Graft function and patient survival at one year were also comparable between groups.

Although recipients treated with rituximab tended to experience fewer rejection episodes and lower rates of elevated donor-derived cell-free DNA, these differences did not reach statistical significance. Notably, rejection events in the rituximab group occurred early after transplantation, whereas most rejection episodes in the no-rituximab group occurred later, suggesting a potential effect of rituximab on the timing rather than the overall incidence of rejection. However, the recurrence rates of preexisting DSA were similar between groups, indicating that rituximab may have limited impact on established humoral memory in this clinical context.

Consistent with prior reports, rituximab use showed trend to increased risk of infection, and infections requiring hospitalization, although these differences were not statistically significant. These findings underscore the importance of balancing potential immunologic benefits against added infectious risk, particularly in patients already receiving lymphocyte-depleting induction therapy.

This is a small, single-center retrospective study, which precluded strong statistical evaluation and making strong recommendations. Another limitation is the lack of CD20, CD19 levels. Moreover, the outcomes, such as acute rejection and infections, could also be influenced by the use of IVIG or plasmapheresis, which was not analyzed in this manuscript. These limitations may cause bias and restrict generalizability.

In summary, our study did not find association between using single dose rituximab in patients with preexisting DSA and overall risk of infectious complications, risk of recurrent or *de novo* DSA or rejection.

## Data Availability

The data that support the findings of this study are available from the corresponding author, MZM, upon reasonable request.
